# Dietary stimulation of the endogenous somatotropic axis in weaner and grower-finisher pigs using medium chain triglycerides and cysteamine hydrochloride

**DOI:** 10.1186/s40104-016-0121-9

**Published:** 2016-10-18

**Authors:** David W. Miller, Zoe Prosser, Edward Y. W. Chee, Christian F. Hansen, Frank R. Dunshea, Bruce P. Mullan, John R. Pluske

**Affiliations:** 1 School of Veterinary and Life Sciences, Murdoch University, Murdoch, WA 6150 Australia; 2 Department of Large Animal Sciences, Faculty of Health and Medical Sciences, University of Copenhagen, Groennegaardsvej 2, 1870 Frederiksberg C, Denmark; 3 Melbourne School of Land and Environment, University of Melbourne, Parkville, VIC 3051 Australia; 4 Department of Agriculture and Food Western Australia, Bentley Delivery Center, Locked Bag 4, Bentley, WA 6983 Australia

**Keywords:** Ghrelin, Growth, Pig, Somatotropin

## Abstract

**Background:**

Three experiments were conducted to examine the overall hypothesis that addition of medium chain triglycerides (MCT) and cysteamine hydrochloride (CSH) into the diets of young and growing pigs would stimulate the endogenous somatotropic axis to improve growth and performance.

**Results:**

In Experiment 1, weaner pigs were given either a 5 d dietary supplement of 5 % MCT (*n* = 8) or a control diet (*n* = 8). MCT increased the plasma concentration of growth hormone (GH; *P* < 0.05) and the GH secretagogue, ghrelin (*P* < 0.05). Additionally, the MCT treatment reduced scouring (*P* < 0.05), maintained villous height in the small intestine (*P* < 0.05) and stabilised daily weight gain (*P* < 0.05), compared to the controls. Experiment 2 compared the effects of 4 levels (0, 1, 3 and 6 % v/v) of MCT supplementation in grower-finisher male pigs, of approximately 35 kg live weight (*n* = 15 per treatment). Blood samples taken after 7 wk of treatment showed that the MCT supplementation increased circulating ghrelin (*P* < 0.001), GH (*P* < 0.01) and insulin (*P* < 0.05) concentrations in a dose-dependent manner. Daily weight gain, feed intake and feed conversion ratio were not affected by the MCT diets. In Experiment 3, 64 female pigs of approximately 60 kg live weight were allocated to one of three dietary treatments: control (*n* = 20); 6 % MCT (*n* = 21); and 70 mg/kg CSH (*n* = 21). After 3 wk of supplementation, the MCT treated pigs had a higher plasma concentration of ghrelin compared to the control and CSH pigs (*P* < 0.05). Plasma concentrations of GH and weight were not affected by any of the dietary treatments.

**Conclusions:**

Evidence is provided in Experiment 1 to support the use of dietary MCT supplements, perhaps acting via stimulation of somatotropic endocrine pathways, to minimise weaning-associated disorders such as slowing of growth and diarrhoea. In Experiments 2 and 3, although there was no effect on weight gain or feed conversion ratio in either experiment, MCT and CSH increased endocrine components of the somatotropic axis.

## Background

Porcine somatotropin (growth hormone; GH), is approved for use in pigs in many countries worldwide [1], although not in the USA or European Union, to improve daily weight gain and promote lean growth. Growth hormone is a protein whose secretion from the somatotrophs of the anterior pituitary gland is regulated by two hypothalamic neurohormones that specifically act to either stimulate (via GH-releasing hormone, GHRH) or inhibit (via somatostatin) the release of GH [2]. The overall effects of GH are to enhance the ability of muscle cells to utilise nutrients, while simultaneously coordinating other physiological processes and tissues (such as adipose tissue), in a manner that supports enhanced lean growth [2].

Previous studies have investigated dietary means of increasing endogenous GH levels. Dietary inclusion of the sulfhydryl compound, cysteamine hydrochloride (CSH), increases GH secretion in rats [3], sheep [4, 5] and fish [6]. The increase in GH secretion is due to the inhibitory effect of CSH on somatostatin release [5]. Dietary supplementations of CSH at 30 mg/kg and 50 mg/kg of feed resulted in significant increases in daily weight gain in finisher pigs, but had no effect on plasma concentrations of GH [7]. A dietary supplementation of CSH at 70 mg/kg of feed in finisher gilts caused an increase in daily weight gain, but GH levels were not measured in this study [8].

Ghrelin, a GH-releasing peptide initially isolated from the stomach of rats [9], stimulates GH release from the anterior pituitary gland [10]. Studies have identified multiple physiological functions for ghrelin in mammals, including GH release, appetite stimulation, cellular proliferation, apoptosis inhibition, and regulation of lipid metabolism and tissue fat distribution in muscle [11–15]. Ghrelin is also reported to be involved in the inhibition of pro-inflammatory cytokine production and gastroprotection against stress-induced gastric damage in rats [16, 17]. Moreover, Salfen et al. [18] showed that ghrelin infusion for 5 d increased GH secretion and concomitantly increased weight gain in weaner pigs. The major active form of ghrelin is a 28-amino acid peptide containing an octanoic (C8:0) fatty acid on the third amino acid (serine) of the peptide [19]. The post-translational C8 modification is essential for biological activity of ghrelin to allow binding to its receptor, which causes the release of GH at the level of the pituitary [19]. It has been reported that ingested medium chain triglyceride (MCT) oil derived from coconuts, which contain high levels of octanoic acid, are directly utilised for the bio-activation of ghrelin in rats [19]. However, no studies to date in pigs have looked at the effect of dietary MCT on somatotropic growth responses and the involvement of bioactive ghrelin levels in the circulation.

Three experiments were conducted to examine the overall hypothesis that addition of MCT and CSH into the diets of young and growing pigs would stimulate the endogenous somatotropic axis to improve growth and performance. The aims of Experiment 1 were to investigate whether the addition of MCT to the diet of weaner pigs would increase the biological activation of ghrelin in the circulation, and if increasing the amount of biologically-active ghrelin would promote GH release. The aim of Experiment 2 was to determine the optimal inclusion rate of MCT into the diets of grower-finisher pigs to investigate somatotropic growth responses, and in Experiment 3, a comparison of the effects of MCT with a CSH dietary supplement for grower-finisher pigs was conducted.

## Methods

These experiments were approved by the Animal Ethics Committees at both Murdoch University (NS1176/06, NS2173/08, NS2253/09) and the Department of Agriculture and Food WA (5-05-33, 2-08-9, 2-09-18) to ensure compliance with the guidelines of the Australian Code of Practice for the Care and Use of Animals for Scientific Purposes. All experiments were conducted at the Medina Research Station, Department of Agriculture and Food WA, Medina, WA, Australia.

### Experiment 1

Twenty four, 21-day-old Large White x Landrace pigs (12 females and 12 intact males), with an average body mass of 5.12 ± 0.24 kg (S.E.M.), were initially allocated into 2 indoor pens (12 pigs per pen) measuring 1.5 m × 1.5 m, and the indoor pens were kept at a constant temperature of 28 °C. The pigs remained in these group pens for 5 d to acclimatize to the post-weaning artificial milk diet. Each pen contained a nipple drinker for water and two milk drinkers.

During the 5 d of acclimatization, the pigs were provided with a commercial artificial milk diet prepared from Pigiplus® (Biostarch P/L, Wendouree. Vic., Australia), as per instructions, with the addition of 50 g of dried bovine colostrum (Nufarm Colostrums, Laverton, Vic., Australia) and 50 mL of a probiotic (Pro B, Dandenong, Vic., Australia) per 8 L of Pigiplus ®. Pigiplus ® contains approximately 14 % fat (as solids when reconstituted), 22 % protein, 43 % lactose and 0.2 % fibre. At 2-h intervals the milk feeders were cleaned and fresh milk added.

After the acclimatization period, 16 pigs (8 males and 8 females) were chosen for the experiment based on their health status (e.g., normal feed intake, normal weight gain, no or minimal scouring). These pigs were separated into single indoor pens of 1.5 m × 0.75 m containing a nipple water drinker and a milk drinker. The pigs were then randomly allocated to the 2 treatments, ensuring that there were equal numbers of each sex, similar sibling relationships and similar group weights between the treatments. The two treatments were a Control group fed a milk replacer diet (*n* = 8; Pigiplus®) and a MCT group fed the same milk replacer with the addition of 5 % (v/v of prepared milk replacer) MCT (MCT Oil; Melrose Laboratories Pty Ltd, Mitcham, Vic., Australia) (*n* = 8) in an homogenous solution. The MCT oil supplement was a refined form of coconut oil containing octanoic acid (C8) (65–75 %), decanoic acid (C10) (25–35 %), and hexanoic acid (C6) (<1 %). The diets were fed at isoenergetic levels with the addition of 4.7 % (v/v) canola oil to the control diet. Treatments continued for 5 d. Live weight and feed refusals were measured daily. During the experiment, the same staff member observed the pigs twice daily, 09:00 h and 15:00 h, for clinical signs of diarrhoea. A pig was recorded as having diarrhoea on a particular day if, at either of the two daily time-points, fresh, loose faeces were adhered to the perineal region of the pig, or if there was observation of loose faeces during defecation.

Immediately prior to the start of the treatments and on d 5, a single blood sample (1 mL) was obtained from all pigs by venepuncture of the superior vena cava. Blood was collected into EDTA vacutainers, immediately centrifuged, an aliquot of the plasma was acidified with 1 % (v/v) HCl for prevention of ghrelin degradation, and all samples stored immediately at -20 °C.

The pigs were euthanized at the start of d 6 with a lethal dose of Valabarb® (Pentobarbitone Sodium, Jurox Ptd Ltd, NSW, Australia), and tissue samples were collected from various regions of the small intestine (duodenum, jejunum and ileum). These tissues were fixed in Bouin’s solution for 6 h before being processed into wax blocks and sectioned at 5 μm using a microtome for histology. Six sections from each tissue, taken at 200 μm intervals, were stained with haematoxylin and eosin. Measurements of villous height and crypt depth were made on the H&E stained slides using the method previously described by Pluske et al. [20]. For each tissue section quantification of villous height and crypt depth was carried out over ten randomly selected fields of view; this approach has previously been shown to be sufficient to stabilise the mean and variance of results [21].

### Experiment 2

From 14 to 22 wk of age, 60 entire male Large White x Landrace pigs (*n* = 15 per treatment) kept in individual grower pens received a standard commercial diet (control), or one of three MCT (MCT Oil; Melrose Laboratories Pty Ltd, Mitcham, Vic., Australia) dietary incorporation rates (1, 3 and 6 %). The diets were adjusted to isoenergetic levels with canola oil, and contained adequate energy, protein and lysine (based on wheat and soybean meal) as lysine has been shown to be essential for a GH increase to have an effect [22]. Live weight and feed refusals were recorded daily. At wk 17 (after 21 d on the dietary treatments), a subgroup of pigs (*n* = 6 per treatment; selected on the basis of growth rate to reflect the group average) had temporary catheters inserted in the vein maxillaris by means of insertion of the catheter through an ear vein (auricularis lateralis or auricularis rostralis). The ear vein cannulation technique was similar to that described in detail by Zanella and Mendl [23]. Catheters were kept patent in between blood samplings by flushing with approximately 1 mL of 5 IU/mL heparin solution in sterile saline (0.9 % w/v Baxter Healthcare, QLD, Australia). The day following cannulation, repeated blood samples (1 mL) were collected at 15-min intervals for 4 h into EDTA-tubes. Initially, 2 mL of blood was withdrawn at each sampling, which was then discarded to avoid dilution of the 1 mL sample with the previous saline flush. The blood tubes were immediately centrifuged, and 200 μL aliquots of the 15 min plasma samples from each animal were pooled, to account for the episodic secretion of GH, and acidified with 1 % (v/v) HCl and immediately stored at -20 °C. Pooled samples were used for hormone analysis.

### Experiment 3

This experiment used 62 crossbred (Large White x Landrace) female pigs that were acclimatised and grown in individual grower pens from an approximate age of 10 wk post partum on a commercial diet until 16 wk of age when they were placed into their dietary treatments. The pigs were weighed, ear-tagged, and allocated at random to 3 treatments consisting of: a control group (commercial diet); a 6 % MCT dietary supplement group (based on the findings of Experiment 2); and a CSH (cysteamine hydrochloride) supplement group, with 21 pigs allocated to each treatment (20 in the control group). The CSH (Porcimax™; Walcom Bio-Chem Co. Ltd, Shanghai, China) was incorporated into the feed at a dose of 70 mg/kg of feed. Live weight was recorded weekly, and feed refusals were recorded daily. At 19 wk post-partum, blood samples were taken from a sub-group of 7 animals per treatment, selected based on growth rates to reflect the group average. The ear vein catherisation and blood sampling protocols used were as described for Experiment 2. At the end of the experiment the pigs were sent to a commercial abattoir (Linley Valley Pork, WA, Australia) once they had reached an appropriate slaughter weight (95 ± 5 kg), where subcutaneous P2 backfat depth, hot carcass weight and carcass dressing percentage were recorded. Depth of backfat at the P2 site (6.5 cm from the midline over the last rib) was measured on the slaughter line using a Hennessy Grading Probe 4 (Hennessy Grading Systems Limited, Auckland, New Zealand).

### Hormone analysis

Plasma collected in Experiments 1, 2 and 3 was analysed for acyl-ghrelin, GH, IGF-1 and insulin. Acyl-ghrelin was determined using an ELISA kit (Acyl Ghrelin (active) EZGRA – 88 K, Lot 1635980, Millipore, Billerica MA USA). The sensitivity of the assay was 25 pg/mL with intra-assay and inter-assay precision of 3.8 % (CV) and 7.5 % (CV), respectively. GH was determined using an ELISA kit (Active GH DSL 10-72100, Lot 891185, Diagnostic Systems Laboratories Inc., Texas USA). The sensitivity of the assay was 0.54 ng/mL with intra-assay and inter-assay precision of 4.1 % (CV) and 9.1 % (CV), respectively. IGF-1 was determined using an ELISA kit (IGF-I DG100, Lot 267244, R & D Systems, Minneapolis USA). The sensitivity of the assay was 26 ng/mL with intra-assay and inter-assay precision of 3.5 % (CV) and 8.1 % (CV), respectively. Insulin was determined using an ELISA kit (Insulin 10-1200-02, Lot 15719, Mercodia AB, Uppsala Sweden). The sensitivity of the assay was 0.062 ng/mL with intra-assay and inter-assay precision of 3.5 % (CV) and 6.9 % (CV), respectively.

### Statistical analyses

Analysis of variance (General Linear Model; Minitab 16, Pennsylvania, USA) was carried out on the data, with treatment and time as the fixed effects and animal (ID, sibling relationships and sex) as random effects. Post-hoc Fisher’s protected least significant difference analysis was used to test for specific differences between treatments at each time point. Differences in the incidence of scouring (diarrhoea) were tested by use of Chi-square analysis.

## Results

### Experiment 1

Over the 5 d of the experiment there was a significant effect of time (*P* < 0.01) but no effect of treatment or the interaction of treatment and time on feed intake. For average daily weight gain (ADG) there was no effect of time or treatment, but there was a significant interaction of treatment and time (*P* < 0.05), and for feed conversion ratio (FCR) there was no effect of treatment or time, but there was a significant interaction of treatment and time (*P* < 0.05). Specifically, the ADG of the control group was increased (*P* < 0.05) compared to the MCT group on d 2 (343 g/d versus 246 g/d) and d 3 (394 g/d versus 235 g/d). The ADG of the MCT group was increased (*P* < 0.05) compared to the control group (240 g/d versus 160 g/d) on d 5, with the control group’s ADG decreasing (*P* < 0.05) by nearly 60 % in the last 2 d of the experiment (Table 1). The FCR of the MCT group was about 45 % higher (*P* < 0.05) and 40 % lower (*P* < 0.01) compared to the control group on d 3 and d 5 of the experiment, respectively. Additionally, it was observed that 4 of the 8 control pigs had overt diarrhea during the last 2 d of the experiment, while none of the MCT-treated pigs were seen to be scouring at any time during the experiment (Chi-square = 5.33, *P* < 0.05).

**Table 1:** Average daily feed intake (ADFI; mL), average daily gain (ADG; g/d), feed conversion ratio (FCR; mL/g) and percentage diarrhoea (% Scour) on each day of treatment for the mixed-sex weaner pigs in the control group (Cont.; *n* = 8) and the medium chain triglyceride supplement (5 % MCT; *n* = 8) dietary treatments

Item	Treatment	Day 1	Day 2	Day 3	Day 4	Day 5
ADFI, mL	Cont.	1489	1798	2024	2213	2492
	MCT	1332	1525	1731	2010	2254
SEM^a^		163	166	182	129	88
ADG, g/d	Cont.	312	343	394	241	160
	MCT	247	246*	235*	251	240*
SEM^a^		40	31	42	37	31
FCR, mL/g	Cont.	4.8	5.2	5.1	9.2	15.6
	MCT	5.4	6.2	7.4*	8.0	9.4**
SEM^a^		0.4	0.5	0.6	0.9	1.1
% Scour	Cont.	0	0	12.5	50	50
	MCT	0	0	0	0*	0*

*= *P* < 0.05 significance between MCT and control treatment

**= *P* < 0.01 significance between MCT and control treatment

^a^SEM = pooled standard error of the mean

**Fig. 1:**
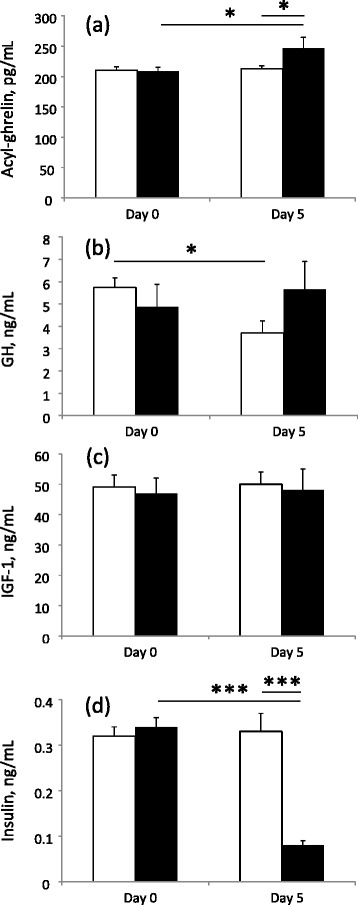
Circulating plasma concentrations of **a** acyl (bioactive) ghrelin, **b** GH, **c** IGF-1 and **d** insulin on d 5 of treatment for 30 days old weaner pigs fed a control (open bars; *n* = 8) or a 5 % dietary MCT supplement (*black* bars; *n* = 8) in Experiment 1. Values are means ± S.E.M. * = *P* < 0.05, *** = *P* < 0.001 significance between MCT and control treatment

Prior to the start of treatments there was no difference between the piglet groups in any of the plasma hormones measured (Fig. 1). Overall, there was no effect of time or treatment on any of the hormones, but there was a significant effect of the interaction of treatment and time for ghrelin (*P* < 0.05), GH (*P* < 0.05) and insulin (*P* < 0.001). Specifically, on d 5 of treatment, the plasma concentration of ghrelin was increased by 16 % (*P* < 0.05) in the in the MCT-treated group compared to the control group. There was no difference in plasma concentration of GH between the treatments on d 5, but there was a significant difference between the change in GH concentration between d 0 and d 5 (*P* < 0.05), with levels decreasing by 34 % in the control group but increasing by 13 % in the MCT group. Plasma concentrations of insulin were 4 times lower in the MCT group on d 5 of treatment (*P* < 0.001), but there was no difference between treatments for the plasma concentration of IGF-1.

**Fig. 2:**
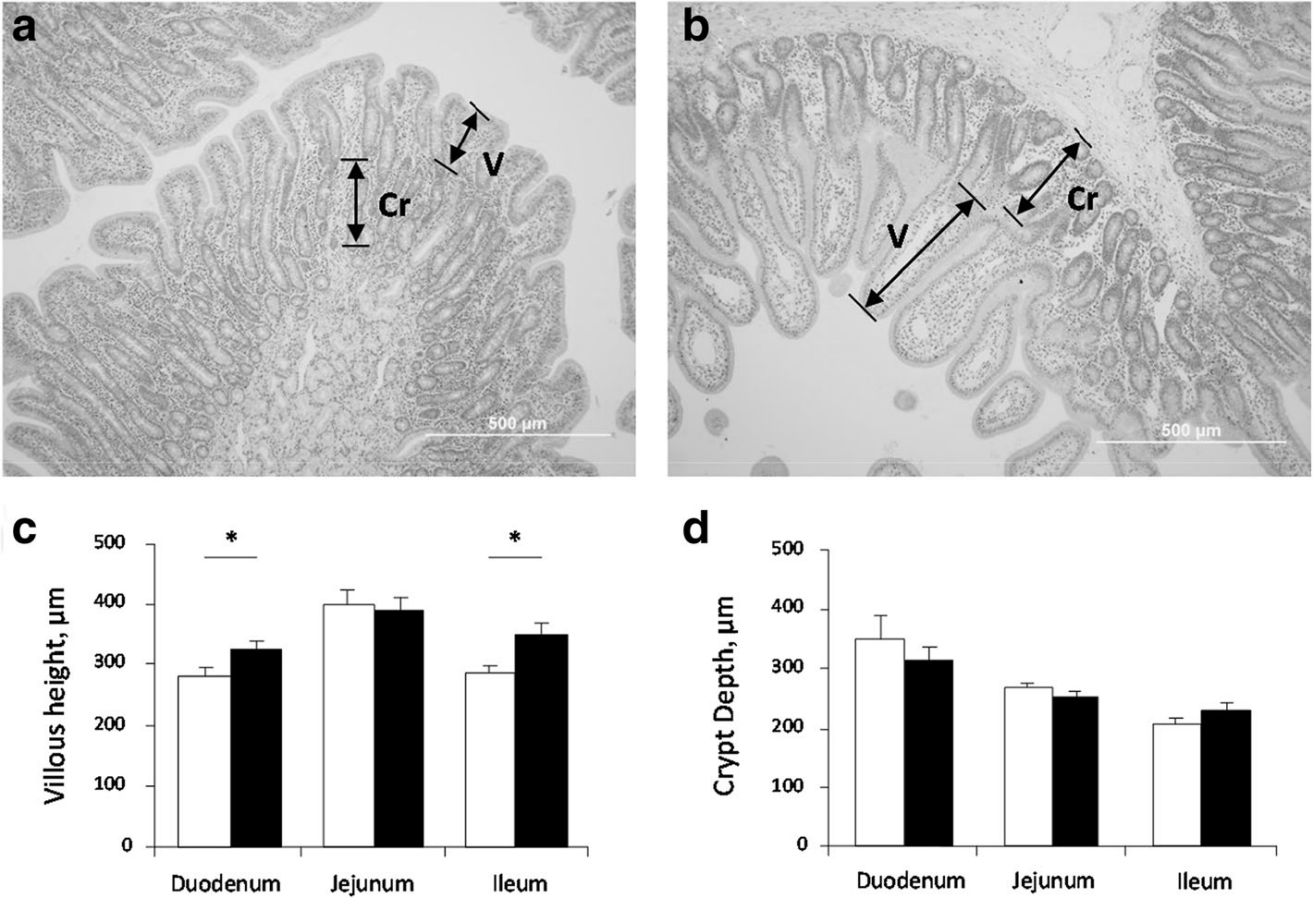
Representative photomicrographs of small intestine (duodenum) indicating differences in villi height (V) and crypt depth (Cr) after d 5 of treatment for 31 days old weaner pigs comparing between the two treatments: control (**a**) and MCT (**b**) in Experiment 1. Scale bar = 500 μm. Corresponding measurements of villous height (**c**) and crypt depth (**d**) of the three sections of the small intestine (duodenum, jejunum and ileum) for the control treatment (*white* bars; *n* = 8 pigs) and the MCT treatment (*black* bars; *n* = 8 pigs). Values are means ± S.E.M. * = *P* < 0.05 significance between MCT and control treatment

Post-mortem histological analysis of gut morphology indicated that villous height in the duodenal and ileal regions of the small intestine was decreased (*P* < 0.05) by about 25 % in the control pigs compared to the MCT-treated group (Fig. 2). There was no difference in crypt depth between the treatment groups.

### Experiment 2

**Fig. 3:**
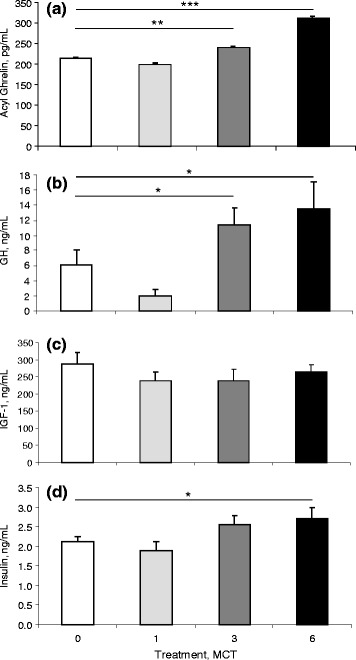
Circulating plasma concentrations of **a** acyl (bioactive) ghrelin, **b** GH, **c** IGF-1 and **d** insulin on d 21 of treatment for 17 weeks old male grower-finisher pigs fed a control (open bars; *n* = 6) or a 1 % (*light gray* bars; *n* = 6), 3 % (*dark gray* bars; *n* = 6), or 6 % (*black* bars; *n* = 6) dietary MCT supplement in Experiment 2. Values are means ± S.E.M. * = *P* < 0.05, ** = *P* < 0.01, *** = *P* < 0.001 significance between each of the MCT treatments individually to the control treatment

Daily weight gain, feed intake and feed conversion ratio were not affected by the inclusion of MCT in the diets, at any of the three levels (1, 3 and 6 %), compared to the control diet during the entire study (Table 2). Plasma hormone analysis of pooled blood samples taken at wk 17 (after 21 d on the dietary treatments) indicated that MCT inclusion increased circulating ghrelin (*P* < 0.001), GH (*P* < 0.01) and insulin concentrations (*P* < 0.05) in a dose-dependent manner, with the 6 % MCT inclusion rate resulting in a 48 % increase in ghrelin concentration (*P* < 0.001), a 130 % increase GH concentration (*P* < 0.05), and a 33 % increase in insulin concentration (*P* < 0.05) compared to controls. MCT inclusion had no effect on circulating IGF-1 concentration (Fig. 3).

**Table 2:** Average daily feed intake (ADFI; kg), average daily gain (ADG; g/d) and feed conversion ratio (FCR; g/g) averaged over treatment period for male grower-finisher pigs (from 14 to 22 wk of age) in the control group (Cont.; *n* = 15), the 1 % MCT supplement (1 % MCT; *n* = 15), the 3 % MCT supplement (3 % MCT; *n* = 15), and the 6 % MCT supplement (6 % MCT; *n* = 15) dietary treatments of Experiment 2

Treatment	ADFI, kg	ADG, kg/d	FCR, g/g
Cont.	2.53	1.088	2.33
1 % MCT	2.61	1.089	2.40
3 % MCT	2.63	1.035	2.54
6 % MCT	2.64	1.084	2.44
SEM^a^	0.06	0.039	0.09

^a^SEM = pooled standard error of the mean

### Experiment 3

Daily weight gain, feed intake and feed conversion ratio were not affected by the inclusion of MCT or CSH in the diets, compared to the control diet, during the entire study (Table 3). There was no difference between treatments in the number of days taken to reach slaughter weight (95 ± 5 kg), or on hot carcass weight or carcass dressing percentage between the three treatments. There was a significant difference between the control and the MCT and CSH treatments for P2 subcutaneous backfat depth, with MCT-fed pigs having a 19 % lower (*P* < 0.01), and the CSH pigs having a 14 % lower backfat depth (*P* < 0.01) compared to the control pigs.

**Table 3:** Average daily feed intake (ADFI; kg), average daily gain (ADG; g/d), feed conversion ratio (FCR; g/g) averaged over treatment period, and days to reach slaughter weight of 95 ± 5 kg from start of experiment (Slaughter; days), subcutaneous P2 backfat depth (P2 Fat; mm), hot carcass weight (HCW; kg) and carcass dressing percentage (Dressing; %) for female grower-finisher pigs (from 16 to 22 wk of age) in the control group (Cont.; *n* = 20), the 6 % MCT supplement (MCT; *n* = 21), and the CSH supplement (CSH; *n* = 21) dietary treatments of Experiment 3

Treatment	ADFI, kg	ADG, kg/d	FCR, g/g	Slaughter, d	P2 Fat, mm	HCW, kg	Dressing, %
Cont.	2.81	1.061	2.65	37.8	13.1	62.4	67.2
MCT	2.67	0.944	2.83	37.1	10.5**	61.2	66.4
CSH	2.79	1.030	2.71	36.2	11.2**	61.8	66.0
SEM^a^	0.05	0.052	0.09	1.7	0.6	0.7	2.6

**= *P* < 0.01 significance between MCT and/or CSH and control treatment

^a^SEM = pooled standard error of the mean

**Fig. 4:**
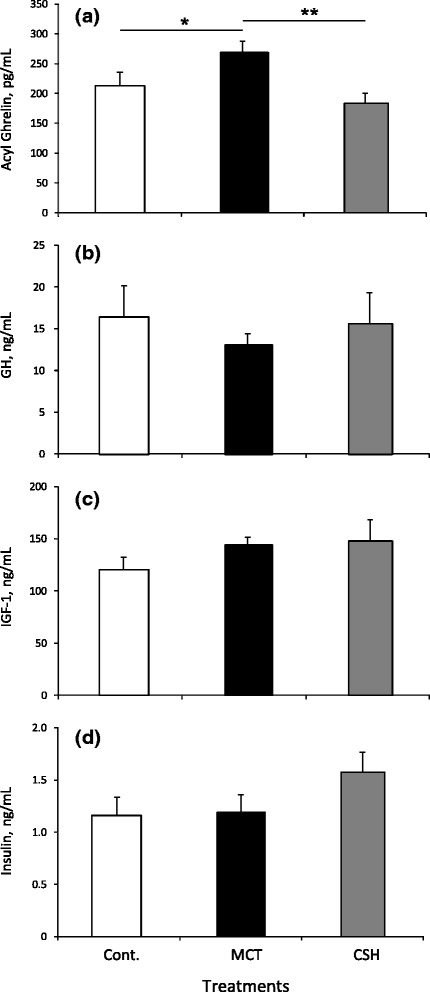
Circulating plasma concentrations of **a** acyl (bioactive) ghrelin, **b** GH, **c** IGF-1 and **d** insulin on d 21 of treatment for 19 weeks old female grower-finisher pigs fed a control (open bars; *n* = 7) a 6 % MCT diet (*black* bars; *n* = 7) or a CSH diet (*gray* bars; *n* = 7) in Experiment 3. Values are means ± S.E.M. * = *P* < 0.05 significnce between MCT and/or CSH and control treatment

There was an overall difference (*P* < 0.05) between treatments in the mean plasma concentration of ghrelin in the pigs. Specifically, MCT-fed pigs had 26 % higher concentrations compared to the control pigs (*P* < 0.05), and 46 % higher (*P* < 0.01) concentrations of ghrelin compared to the CSH pigs (Fig. 4). There was no difference in GH, IGF-1 or insulin concentrations between the treatment groups.

## Discussion

### Experiment 1

This study found that whilst 5 % MCT oil supplemented for 5 d had no effect on improving feed intake or growth, it did appear to prevent the diarrhoea-associated decline in growth rates seen in the control pigs. There was also evidence to indicate that dietary MCT could be used to increase the plasma concentration of bioactive ghrelin. The present findings agree with Nishi et al. [19] who demonstrated in rats that a 5 % MCT-oil supplement increased the plasma concentration of acylated (bioactive) ghrelin without changing total ghrelin levels. Octanoic acid in the diet of the MCT-fed pigs may be attaching to the third amino acid (serine) of the des-acyl ghrelin (inactive) peptide, a modification that allows binding to the ghrelin peptide to its receptor, and hence is essential for biological activity [24]. Salfen et al. [18] showed that ghrelin infusion for 5 d positively influenced weight gain and concomitantly increased GH secretion in weaner pigs. Although there was an indication in the present study that circulating GH concentrations were affected, the response wasn’t of the same magnitude as found by Salfen et al. [18]. This may be because the modest increase in bioactive ghrelin levels, by about 16 % in the MCT-fed pigs, wasn’t of the same magnitude of increase seen with the infusion that caused a 25-fold increase in total ghrelin levels. Although these two studies measured different forms of the ghrelin molecule, the difference in magnitude of response is noteworthy. Increasing the amount of MCT oil in the diet to achieve higher bioactive ghrelin levels may be problematic as high oil contents could result in nutritionally unbalanced diets. Moreover, a study in neonatal pigs found that MCT-oil supplements could cause narcotic effects [25] that may cause young pigs to be less vigorous and thus increase mortality.

There was a significant decrease in circulating insulin concentrations in the MCT-fed weaner pigs. The effect of MCT supplementation on insulin secretion in adult human and rodent studies is equivocal [26], but the majority reports a slight hyperinsulinemic response [27–30]. In the pig, weaning is usually associated with a decrease in insulin due to the dietary energy deficit [31]. However, this hypoinsulinemic effect is quickly attenuated once the weaner pig starts eating [32–34]. Insulin is very important to the neonatal pig at this stage of its life as it ensures rapid skeletal muscle protein deposition [35]. As the MCT diet in the present study was fed at isoenergetic levels to the control diet, the reason why insulin levels decreased in the MCT supplemented weaner pigs is unknown. It is known though, in humans, that MCT-based diets produce lower post-prandial glucose increases than long chain triglyceride (LCT)-based diets [36, 37], and it has been suggested that MCT diets improve insulin sensitivity in human and rodent studies [38, 39].

The MCT-fed pigs had no diarrhea whilst half of the control pigs were scouring by the end of the 5 d treatment period. The occurrence of scouring in the control pigs was also associated with a decline in growth rates. When we examined the small intestines of the pigs we found reduced height of the villi in the duodenal and ileal regions of the control pigs compared to the MCT treated pigs. Price and colleagues also showed a beneficial effect of dietary MCT on the intestinal villi in MCT-fed weaner pigs compared to LCT-fed pigs [40]. The period following weaning is generally characterised by a high incidence of intestinal disturbances with diarrhoea and depression of growth performance being common [39]. Weaning is often coincidental with a reduction in villous height in the intestine [19, 41–49] that leaves fewer and less-differentiated enterocytes on villi available for the digestion of nutrients [50, 51]. A concomitant reduction in the capacity of the small intestine to absorb xylose [51–53], alanine [46, 50] and electrolytes [54] has also been reported after weaning. Studies have identified physiological functions for ghrelin apart from stimulation of the somatotropic axis, including cellular proliferation, apoptosis inhibition and boosting the immune system [55, 56]. Therefore, it may be plausible to postulate that the ghrelin-stimulating effect of the MCT diet in the present study may have been one mechanism to explain the decrease in weaning-associated problems and improved intestinal functional capacity, although further studies with larger sample sizes would be warranted to draw firm conclusions about the effect of MCT on scouring.

### Experiment 2

This study demonstrated that a 6 % MCT dietary incorporation for grower/finisher pigs was optimum (compared to 1 and 3 %) for increasing the bio-activation of ghrelin and increasing circulating GH concentrations. However, there was no effect on growth performance in this study. A possible reason for the lack of effect may have been that the level of GH stimulation by the 6 % MCT dietary treatment (about 14 ng/mL, a 2-fold increase compared to the controls) may not have been sufficient to significantly affect growth. Hansen et al. [57] found that a daily pST (porcine somatotropin) injection in grower pigs that raised endogenous GH concentrations to about 14 ng/mL had no effect on muscle protein accretion, whereas Klindt et al. [58] found that pST implants that raised endogenous GH concentrations in castrated male pigs to about 30 ng/mL caused a 22 % increase in average daily gain. Indeed, the lack of effect of MCT supplementation on IGF-1 levels in the present study also suggests that GH stimulation was only modest.

There was a dose-dependent increase in circulating insulin concentrations in the MCT-fed pigs. As mentioned above for Experiment 1, the effect of MCT supplementation on insulin secretion in other studies is equivocal [26]. In studies where insulin secretion has been stimulated it has been ascribed to an indirect effect of increased ketogenesis on pancreatic function [29]. Another possible mechanistic pathway may be provided by the insulin-stimulating effect circulating ghrelin has on pancreatic cells in vitro [59], however studies into the role of ghrelin in altering insulin secretion are also equivocal [60]. It is well known that exogenous pST treatment induces a state of insulin resistance in pigs resulting in decreased glucose uptake by adipose tissue [61, 62] and resultant hyperinsulinemia [63].

### Experiment 3

The hypothesis that addition of 6 % MCT or CSH to the diets of grower/finisher pigs would increase endogenous GH levels, increase the rate of weight gain and improve feed efficiency, was not supported. There was no effect of the MCT or CSH diet on plasma GH concentrations, average daily weight gain or feed intake. However, the MCT diet did increase circulating levels of active ghrelin and both the MCT and CSH diets decreased the P2 backfat depth. The reason for the minimal effect on the somatotropic system is not known, however the lack of GH stimulation and the concomitant lack of effect on weight gain and feed intake could be gleaned from the study by Etherton et al. [64], who found that increases in plasma GH concentrations to about 25 ng/mL following pST injection are needed to increase weight gain. Possible reasons for the lack of a GH response in the MCT and CSH supplemented animals include: 1) insufficient stimulation of bioactive ghrelin by MCT and insufficient suppression of somatostatin by CSH to cause an increase in GH levels; 2) insufficient numbers of animals to pick up a modest increase in GH and/or growth stimulation; or 3) that the MCT and CSH treatments may have initially increased GH levels, but this effect wasn’t sustained by the day of blood sampling. In this context, the increase in daily gain in response to CSH observed by Dunshea [8] was transient in nature and most apparent in the first 2 wk of treatment.

As was postulated for Experiment 1 (Section 4.1), the modest stimulation of the somatotropic axis in the MCT-fed pigs could be due to the modest increase in active ghrelin levels. In Experiments 2 and 3 the ghrelin levels only increased to about 260 ng/mL in the 6 % MCT-treated groups. This level may be insufficient to fully stimulate the somatotropic axis. However, the decrease in GH levels were attenuated in Experiment 1 with weaner pigs (after 5 d of dietary MCT treatment) and increased in Experiment 2 with male pigs at 17 wk of age (after 3 wk of dietary MCT treatment), but not in Experiment 3 with female pigs at 19 wk of age (after 9 wk of dietary MCT treatment). There are human studies indicating age and gender differences in ghrelin’s actions [65, 66], so it is possible that the differences in the present study could be attributed to differences such as these.

The lack of somatotropic response to CSH in the present study is difficult to explain. While Yang et al. [7] found that dietary supplementation of CSH, at lower inclusion rates to the present study, caused significant increases in daily weight gain in finisher pigs, they also had no effect on plasma concentrations of GH. However, Dunshea [8] found that dietary supplementation of CSH, at the same rate as the present study and with similar age and gender pigs, caused an increase in daily weight gain. As somatostatin was not measured in either the study by Dunshea [8] or in the present study, it is uncertain whether the dose of CSH used actually suppressed somatostatin secretion, the postulated mode of its action [5]. An area of research that needs to be investigated is the dose response to CSH, as there appears to be 10-fold range of doses being investigated and it may be that down-regulation of response [8] is more rapid at higher doses as used in the present study.

There was a significant reduction in P2 backfat depth with the MCT and CSH treatments. As stated above, this was not accompanied by an increase in GH levels, so a GH-stimulated increase in lipolysis, as suggested by researchers using pST injections [67], appears not to account for this effect. There are a number of possibilities for the reduction in P2 backfat. Firstly, the reduction in adiposity may be a result of the small decrease in food intake in the CSH and MCT pigs. However, as this was not a significant effect it is unlikely to be responsible for the 14–19 % decrease in P2 backfat depth. Secondly, there may have been a direct effect of MCT on adipogenesis or lipolysis. Well controlled studies in rats fed MCT via a gastrostomy tube showed that it caused a decrease in weight, and that this decrease was due to decreased fat deposition and not loss of lean body mass [68, 69]. The authors of these studies attributed the effect to an enhanced metabolic rate. Thirdly, the bioactive ghrelin increase in the MCT group may have directly stimulated lipolysis. Ghrelin is capable of stimulating lipolysis in rodents and dairy cows [67, 70]. Finally, the MCT and CSH treatments may have increased the sensitivity of the adipose tissue to the somatotropic/adrenergic system [71]. GH is a chronic, homeorhetic effector of adipose tissue metabolism that has been shown to enhance the lipolytic response to an adrenaline challenge in lactating cows, growing steers, and growing pigs [71]. It is possible that in our study, even though circulating GH levels were not altered, adrenaline sensitivity of the adipose tissue may have been altered leading to the decline in P2 depth. Finally, Yang and colleagues [7], studying CSH effects in pigs, also found a decrease in backfat without effects on GH. They stated that the CSH effects on backfat could have been mediated through the anabolic effects of glucagon and thyroid hormone, which increased in their study, on the skeletal muscles and catabolic effects on the adipose tissue. These mechanisms warrant further investigation.

## Conclusions

Evidence was provided in Experiment 1 showing that dietary MCT offers some promise as a means to bio-activate ghrelin, being the possible mechanism that led to the enhanced villous height observed. Although the experimental period was too short to examine longer-term effects of MCT on production measures, there was some suggestion that MCT reduced diarrhoea in the milk-fed pigs and reduced the post-weaning decline in GH levels. Incorporating MCT into diets (milk formulas, solid diets) of pigs might be a useful aid for the pig industry in terms of decreasing weaning-associated problems.

In Experiments 2 and 3 there was no effect of MCT on growth performance or stimulation of the somatotropic axis, apart from stimulation of circulating bioactive ghrelin concentration. There was also no effect of CSH on growth performance or stimulation of the somatotropic axis, indicating that either the design was insufficient to pick up modest changes in GH levels and weight gain, or that the effects of MCT and CSH on the decrease in backfat that was seen are acting independently of somatotropic action.

## Abbreviations

ADFI: Average daily feed intake
ADG: Average daily gain
C10: Decanoic acid
C6: Hexanoic acid
C8: Octanoic acid
CSH: Cysteamine hydrochloride
FCR: Feed conversion ratio
GH: Growth hormone
GHRH: Growth hormone releasing hormone
IGF-1: Insulin-like growth factor 1
MCT: Medium chain triglycerides
pST: Porcine somatotropin
